# An AC-type element mediates transactivation of secondary cell wall carbohydrate-active enzymes by PttMYB021, the *Populus* MYB46 orthologue

**DOI:** 10.1186/1753-6561-5-S7-O40

**Published:** 2011-09-13

**Authors:** Ines Ezcurra, Camilla Johansson, Prashanth Tamizhselvan, Anders Winzell, Henrik Aspeborg

**Affiliations:** 1 KTH Biotechnology, 106 91 Stockholm, Sweden

## Background

The transcription factor MYB46, together with its redundant paralog MYB83, regulates expression of secondary cell wall biosynthesis genes in Arabidopsis [reviewed in 1].

We isolated the Populus MYB46 ortholog, PtxtMYB021 from hybrid aspen (Populus tremula x tremuloides) [2]. Transiently expressed MYB021 transactivated gene promoters of Populus xylan-active enzymes GT43A, GT43B and Xyn10A. Analysis of conserved motifs within these promoters identified the sequence CCACCAAC, which is similar to the AC elements mediating transactivation by MYB transcription factors during lignin biosynthesis, and we showed that this motif is enriched in xylem-specific carbohydrate active enzyme (CAZyme) promoters.

## Methods

To establish whether the AC-type motif in CAZyme promoters is important for their function, we analyzed loss-of-function and gain-of-function GUS reporter constructs of the GT43A promoter in a transient transcription assay, by co-infiltration with a 35S:MYB021 effector in Nicotiana benthamiana leaves.

## Results and discussion

**Figure 1:**
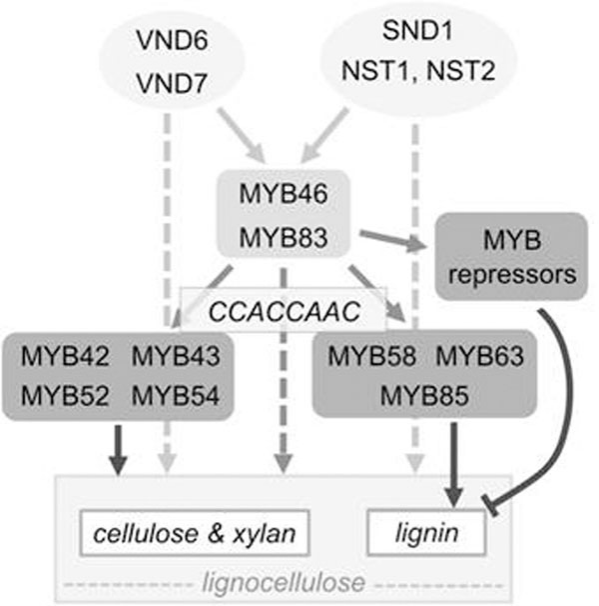
Regulatory networks in lignocellulose synthesis in angiosperms, shown using Arabidopsis gene nomenclature, involve feed-forward loop circuits. Dashed arrows highlight direct target gene activation by higher hierarchy regulators. White boxes at the bottom indicate target gene programs. For simplicity, some regulators, including ASL19, MYB103 and KNAT7, are omitted. Adapted from [1] and references therein.

We show that mutation of the AC-type element in the GT43A promoter abolishes transactivation by MYB021, whereas an AC multimer confers MYB021 transactivation to a minimal 35S promoter. Others have shown that the recombinant Eucalyptus grandii MYB46/MYB83 ortholog, EgMYB2, directly binds the AC-motif variant CCACCTACC found in the EgCCR gene promoter [3], suggesting that MYB021 directly binds the AC-type motif. We propose that, in angiosperms, the AC-type regulatory element mediates MYB46's transactivation of its target genes. Further, MYB46 activates lignocellulose synthesis in a feed-forward loop with downstream MYBs, involving both direct activation of downstream MYBs and joint regulation of downstream secondary cell wall synthesis targets (Fig. 1). Recent results show direct activation of downstream lignocellulose synthesis targets by higher hierarchy NAC domain regulators [4,5].

Interestingly, the downstream MYB regulators of lignin biosynthesis MYB58 and MYB63 bind the AC element but reportedly do not regulate CAZyme-mediated secondary cell wall polysaccharide biosynthesis [6]. Other downstream MYBs that bind variants of the AC element are the EAR domain lignin repressors, such as EgMYB1 and ZmMYB31 (Table 1). Then, binding of the distinct lignocellulose synthesis MYB transcription factors to particular AC-element variants may be mediated by motif sequence, motif context and/or interactions with cofactors. Also, some AC element variants overlap extensively with each other and with the AC-type element defined by us (Table 1), so it remains to be established whether they are all representatives of one identical core sequence motif, as previously proposed [7]. Establishing the exact sequence requirements for AC-element recognition by distinct MYBs will facilitate elucidation of lignocellulose-related regulatory networks in different species, through bioinformatic analysis [8].

**Table 1:** Sequence overlap in AC motifs.

Species	Genbe	Motif name	Motif sequence	MYB protein^†^	Reference
***Phaseolus***	**PAL2**	**AC-I**	CCCACC**T**ACC	**n.d.**	[9]
***Phaseolus***	**PAL2**	**AC-II**	CCACCAACCC...	**n.d.**	[9]
***Petroselinum***	**4CL1**	**AC-II**	C**T**CACCAACCC	**n.d.**	[10]
***Populus***	**CCoAOMT**	**AC-II**	C**T**CACCAACCC...	**n.d.**	[7]
***Eucalyptus***	**EgCCR**	**MBSIIG**	**c**CACC**T**ACC	**EgMYB2^§^**	[3]
***Eucalyptus***	**EgCCR**	**MBSIIG**	**c**CACC**T**ACC	**EgMYB1**	[11]
***Arabidopsis***	**4CL1**	**AC-II**	**t**CACCAAC	**MYB58/63**	[6]
***Zea***	**ZmCOMT**	**AC-II**	**t****c**ACCAAC	**ZmMYB31**	[12]
***Populus***	**GT43A**	**AC-type**	CCACCAAC	**PttMYB021^§^**	[2]
			**** **		

In motif sequences, lowercase indictates ases outside the defined motif, and asterisks highlight invariant bases. Similarity to the GT43A AC-type elements is underlined. ^†^Indicates which MYB protein binds the motif. ^§^Ortholog of MYB46.

## Conclusions

The AC regulatory element, here preliminarily redefined as the sequence CCACCAAC, is a true “lignocellulose synthesis response element”, mediating MYB46-dependent transactivation of the whole secondary cell wall gene program. This knowledge will enable inference of MYB46-centered regulatory networks in different species, through bioinformatic analysis.
